# Attosecond precision multi-kilometer laser-microwave network

**DOI:** 10.1038/lsa.2016.187

**Published:** 2017-01-13

**Authors:** Ming Xin, Kemal Şafak, Michael Y Peng, Aram Kalaydzhyan, Wen-Ting Wang, Oliver D Mücke, Franz X Kärtner

**Affiliations:** 1 Center for Free-Electron Laser Science, Deutsches Elektronen-Synchrotron, 22607 Hamburg, Germany; 2 Department of Physics, University of Hamburg and the Hamburg Center for Ultrafast Imaging, 22761 Hamburg, Germany; 3 Research Laboratory of Electronics, Massachusetts Institute of Technology, Cambridge, MA 02139, USA

**Keywords:** attosecond photonics, attosecond timing metrology, hard-X-ray free-electron laser, laser-microwave network, timing distribution

## Abstract

Synchronous laser-microwave networks delivering attosecond timing precision are highly desirable in many advanced applications, such as geodesy, very-long-baseline interferometry, high-precision navigation and multi-telescope arrays. In particular, rapidly expanding photon-science facilities like X-ray free-electron lasers and intense laser beamlines require system-wide attosecond-level synchronization of dozens of optical and microwave signals up to kilometer distances. Once equipped with such precision, these facilities will initiate radically new science by shedding light on molecular and atomic processes happening on the attosecond timescale, such as intramolecular charge transfer, Auger processes and their impacts on X-ray imaging. Here we present for the first time a complete synchronous laser-microwave network with attosecond precision, which is achieved through new metrological devices and careful balancing of fiber nonlinearities and fundamental noise contributions. We demonstrate timing stabilization of a 4.7-km fiber network and remote optical–optical synchronization across a 3.5-km fiber link with an overall timing jitter of 580 and 680 attoseconds root-mean-square, respectively, for over 40 h. Ultimately, we realize a complete laser-microwave network with 950-attosecond timing jitter for 18 h. This work can enable next-generation attosecond photon-science facilities to revolutionize many research fields from structural biology to material science and chemistry to fundamental physics.

## Introduction

**Figure 1:**
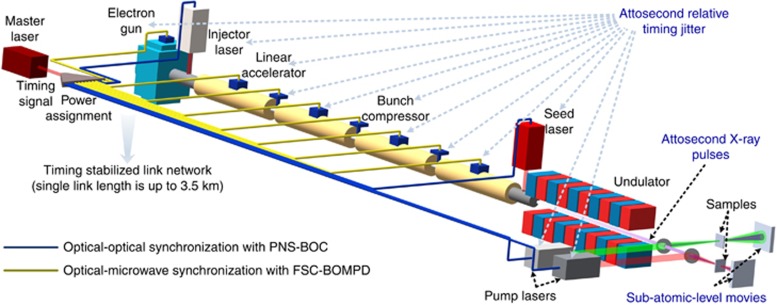
Layout of a timing distribution system for next-generation free-electron lasers. A single timing stabilized link length is up to 3.5 km, which corresponds to the length of the European XFEL, the largest photon-science facility in the world close to completion. PNS means polarization-noise-suppressed and FSC means free-space-coupled.

The quest for isolated attosecond hard-X-ray pulses has markedly intensified over the past few years^1^ with the first observation of intramolecular charge transfer^2^ and the discovery of ultrafast Auger processes altering the chemistry of matter on an attosecond timescale^3, 4^. Next-generation photon-science facilities such as X-ray free-electron lasers (XFELs; e.g., the European XFEL^5^, Free Electron Laser Radiation for Multidisciplinary Investigations (FERMI)^6^, SwissFEL, Linac Coherent Light Source (LCLS)^7^ and LCLS II^8^) and intense laser beamline facilities (e.g., the Extreme Light Infrastructure (ELI)^9^) are emerging worldwide with the goal of generating sub-femtosecond X-ray pulses with unprecedented brightness to capture ultrafast chemical and physical phenomena with sub-atomic spatio-temporal resolution^10, 11^. These facilities, however, cannot fulfill this long-standing scientific dream without a high-precision timing distribution system. As illustrated in Figure 1, the critical task of timing distribution is to synchronize various microwave and optical sub-sources across multi-kilometer distances required for seeded FELs and attosecond pump-probe experiments. So far, there have been no reports demonstrating a synchronous laser-microwave network that permits attosecond precision across such distances. Hence, attosecond-precision synchronization is a major obstacle that prevents attosecond-resolution photon science at hard-X-ray wavelengths.

Two basic timing distribution schemes have been reported to date. The first scheme uses traditional microwave signal distribution via amplitude modulation on a continuous-wave optical carrier^12^. This scheme solely depends on electronic phase-locking techniques and so far has not delivered better than ~100-fs root-mean-square (RMS) jitter facility wide^13^ due to low-timing discrimination with microwaves and high noise floor at photodetection. The second scheme^14, 15^, which is pursued in this paper, uses ultralow-noise pulses generated by a mode-locked laser^16, 17^, as the timing signal to synchronize optical and microwave sources using balanced optical cross-correlators (BOCs)^18^ and balanced optical-microwave phase detectors (BOMPDs)^19^, respectively. In contrast to techniques used in frequency metrology^20, 21, 22, 23, 24^, this approach eliminates the need for additional laser frequency combs at each end station, since it utilizes the ultrashort optical pulses directly as time markers for precision timing measurements and features orders-of-magnitude higher timing stability. While this pulsed scheme has breached the 10-fs precision level^25, 26, 27^, realizing and maintaining attosecond precision requires new metrological devices and better physical understanding of optical pulse shaping in fiber transmission, and its impact on optical/microwave measurements at the fundamental level. This advanced level of physical/technical comprehension is a prerequisite to unfold the full potential of next-generation attosecond photon-science facilities.

To this matter, we have thoroughly analyzed pulse propagation effects in the fiber link and systematically eliminated noise limitations in the whole system to develop a new pulsed timing distribution system. Here we present the first demonstration of a laser-microwave network with attosecond timing precision, which corresponds to a >10 × improvement in timing stability compared with the previously published results^27, 28^, and satisfies the imperative and challenging synchronization requirements for next-generation photon-science facilities.

## Materials and methods

### Simulation model

We developed a numerical model to simulate pulse timing jitter during nonlinear pulse propagation in the fiber link. In this model, the master equation of a fast-saturable-absorber mode-locked laser is solved using the fourth-order Runge–Kutta in the interaction picture (RK4IP) method^29^. Laser timing jitter is generated by adding amplified spontaneous emission noise during each iteration of RK4IP, whose amount corresponds to the measured jitter of the master laser^26^. The pulse train is centered at 1550 nm with 170-fs pulse width and 216-MHz repetition rate. Self-phase modulation, self-steepening and Raman effect are considered in the link. Both the nonlinear Schrödinger equation for the link transmission and the pulse-coupled field equation for second-harmonic generation in the BOC are solved using the split-step Fourier method with an adaptive step length. The BOC characteristic (i.e., the BOC output voltages with respect to the initial delay of the two input pulses) is calculated for each round-trip link pulse against a new laser reference pulse. The timing offset of the zero-crossing position in the BOC characteristic is identified as timing error.

**Figure 2:**
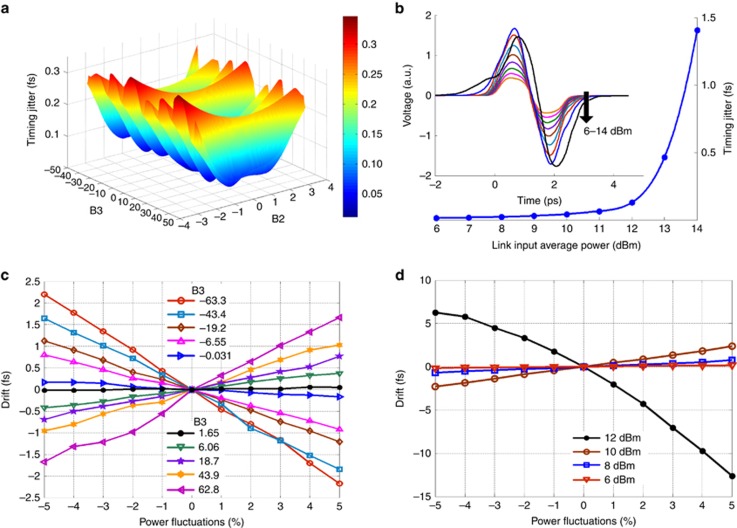
Analysis of link-induced timing jitter and drift. A 216-MHz, 170-fs pulse train from a mode-locked laser, with an integrated timing jitter of 0.3 fs >1 kHz (ref. 26), is sent into a 3.5-km PM fiber link with an erbium-doped optical fiber amplifier and a fiber mirror near the link output. Relative timing jitter between the round-trip pulse train and the original pulse train is calculated in **a** and **b** for frequencies >1 kHz. Timing drift errors introduced by the link stabilization feedback are shown in **c** and **d**, which are related to the environmental fluctuations occurring on slow timescales <10 Hz. (**a**) Link-induced Gordon–Haus jitter due to residual link dispersion. B2: the link’s residual SOD normalized by the SOD of 1-m standard polarization-maintaining fiber PM 1550; B3: the link’s residual TOD normalized by the TOD of 1-m PM 1550 fiber. The ripples along the B3 axis are mainly due to pulse shaping induced by phase fluctuations through TOD. (**b**) Link-enhanced timing jitter due to fiber nonlinearities and the corresponding BOC characteristics at each input power level. The timing sensitivity increases with input power. This jitter caused by fiber nonlinearity needs to be carefully minimized in practice because it easily reaches femtosecond level before a visible distortion of the BOC characteristic can be observed. (**c**) Timing drift induced by link input power fluctuations for different B3 values; for each curve, the input power level is +8 dBm and B2=−0.13. (**d**) Timing drift induced by link input power fluctuations for different input power levels; for each curve, B2=−0.13 and B3=18.7.

To calculate link-enhanced excess jitter (Figure 2a and 2b), the simulation is repeated for a train of laser pulses in the presence of pulse timing jitter. The RMS of the timing errors from all the BOC characteristics is calculated to obtain the overall link-enhanced excess jitter. To calculate the power fluctuation-induced drift (Figure 2c and 2d), only one pulse is simulated in the absence of pulse timing jitter, since this source of timing error is deterministic.

### BOC characteristics

Two methods are used to experimentally characterize the timing sensitivity of a BOC. The first method is for the case, where the two input pulse trains in the BOC have the exact same repetition rate. The relative delay between the pulse trains is swept with a motorized delay stage, while the response voltage of the BOC is recorded with a data acquisition card. The slope of the measured BOC characteristic at its zero-crossing is the timing sensitivity. In the second case, two laser input pulse trains with different repetition rates are combined in a BOC, a train of BOC characteristics is generated at the BOC output. One can simultaneously record a BOC characteristic on an oscilloscope and measure the instantaneous repetition-frequency difference (RFD) between the lasers with photodetectors and electrical mixers. The real timescale of the BOC characteristic can be calibrated by multiplying the oscilloscope timescale with the ratio of the RFD to the laser repetition rate. Coarse frequency tuning is performed in advance to ensure a small RFD so that the BOC characteristic is not limited by the balanced photodetector (BPD) bandwidth.

### BOMPD characteristics

The phase sensitivity of the BOMPD can similarly be measured using a free-running laser and a microwave oscillator. Due to aliasing during electro-optic sampling, the effective frequency difference between the oscillators is *f*_beat_=*f*_RF_ mod *f*_rep_, where *f*_RF_ is the frequency of the radio-frequency (RF) oscillator and *f*_rep_ is the fundamental repetition rate of the laser. The BOMPD output voltage signal will be a train of BOMPD response characteristics with a repetition frequency equal to this frequency difference *f*_beat_. One can record this frequency difference and a single BOMPD characteristic simultaneously with an oscilloscope. The oscilloscope timescale multiplied by the angular frequency difference (2*πf*_beat_) represents the phase error between the optical and RF signal relative to the RF signal frequency. The BOMPD phase sensitivity is defined as the slope of the BOMPD characteristic at its zero-crossing in units of V rad^−1^. The timing sensitivity in units of V s^−1^ is obtained by further multiplying the phase sensitivity by the RF oscillator angular frequency (2*πf*_RF_).

### Other measurement methods

The noise floors of all BOCs in the experiments are limited by the detector electronic noise, since the signal power from second-harmonic generation is relatively low. The feedback timing precision of the BOC is calculated as the integrated RMS noise voltage of the BPD within the locking bandwidth, calibrated by the BOC timing sensitivity.

The long-term drift data in Figure 4e are measured by filtering the out-of-loop signal with a 1-Hz low-pass anti-alias filter and recording with a data acquisition card at a 2-Hz sampling rate. The jitter spectral density data in Figure 4g are direct baseband power spectrum measurement of the BOC/BOMPD output on a signal source analyzer. In Figure 4h, the data >1 Hz is the integration of the timing jitter spectrum in Figure 4g; the data <1 Hz is integrated using the Fourier transform of the drift data in Figure 4e.

## Results and discussion

### Link-induced timing jitter and drift

In the pulsed timing distribution approach (Figure 1), an optical pulse train with ultralow jitter (timing signal) is generated from a mode-locked laser (master laser), and distributed through polarization-maintaining (PM) dispersion-slope-compensated fiber links in a star network topology. At the end of each link, an output coupler partially reflects the timing signal back toward the link input. The timing offset between the returning link pulse and a new pulse from the master laser is measured with a BOC. The error voltage signal from the BOC is fed back to a variable delay line in the link path to compensate for any detected timing errors. Using this feedback scheme, various environmental fluctuations, including mechanical stress, acoustics and temperature imposed on the link can be significantly corrected for. The fundamental limits to this noise suppression scheme are set by the inherent laser noise, BOC detection noise floor, reference path noise and link-induced noise. Of these limitations, the link-induced noise will dominate and prevent optimum link performance if not properly accounted for.

On the basis of our numerical model, residual link dispersion and nonlinearities add considerable excess jitter in the high-frequency range >1 kHz even in the absence of environmental noise. First, pulse center-frequency fluctuations are coupled to timing jitter via residual second-order dispersion (SOD) and third-order dispersion (TOD; Figure 2a). This jitter contribution, often called Gordon–Haus jitter^30^, can amount to 0.1 and 0.3 fs for uncompensated SOD equivalent to 2 and 3 m of standard PM fiber, respectively. Second, spontaneous emission noise is coupled to timing jitter and its impact is further enhanced by link nonlinearities (Figure 2b). This jitter is bounded at 0.13 fs for average power levels <+12 dBm (corresponding pulse peak power *P*_peak_=430 W), but escalates to 1.4 fs at +14 dBm (*P*_peak_=682 W). Since these excess jitter contributions can transfer to the link output through the feedback loop (Supplementary Fig. S2d), fiber-link dispersion and nonlinearities must be minimized to achieve attosecond link stability.

Moreover, link power fluctuations on slower timescales can similarly introduce timing errors that degrade link stability. Long-range compensation for link stabilization is performed by a free-space motorized delay line (MDL) with long delay arms; e.g., a 10-cm range is required to correct for ±1.5-K temperature change in a 3.5-km link. Movement of the delay stage introduces inevitable beam misalignments that cause link power fluctuations. These fluctuations induce temporal shifts in the pulse center-of-gravity through a composite effect of residual SOD, TOD and nonlinearity (Supplementary Equation S28). Although a center-of-gravity shift appears as a deterministic shift in the zero-crossing position of the in-loop BOC characteristic, the link stabilization feedback will unknowingly track this shift and introduce it into the link path, causing a timing error at the link output. Simulations are performed using typical values observed in the experiment. Figure 2c shows that residual TOD can induce timing errors up to 5 fs for +8-dBm link power with ±5% fluctuations. Figure 2d indicates that +10-dBm link power is the threshold before significant amplitude-to-timing conversion occurs due to severe nonlinear pulse distortions and may result in 4 fs of timing error from ±5% power fluctuations. Link power variations and residual TOD must be minimized to achieve long-term attosecond precision.

### Laser-microwave network

**Figure 3:**
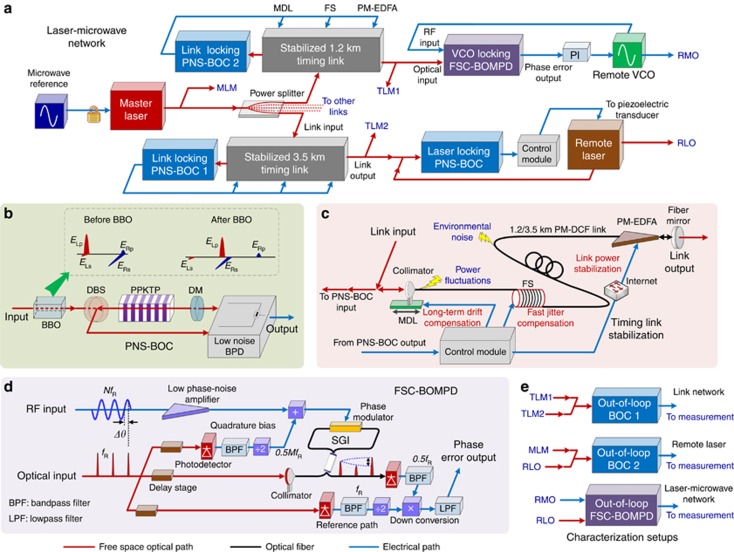
Experimental setup. (**a**) Schematic of the laser-microwave network. (**b**) All BOCs consist of a single 4-mm-long periodically poled KTiOPO_4_ (PPKTP) crystal operating in a double-pass configuration with appropriate dichroic beam splitter and mirror (DBS and DM). Ideally, the input pulses (*E*_Lp_ and *E*_Rs_) are aligned along the two principal axes of the type-II PPKTP crystal for maximum second-harmonic generation. Due to finite polarization extinction ratios of the optical elements upstream from BOCs, there will be undesired pulse components (*E*_Ls_ and *E*_Rp_) projected along the undesired polarization axes. In PNS-BOC, a linear material with large birefringence is put before the BOC. This material adds a significant delay to the erroneous pulses such that they do not overlap and interfere with the second-harmonic generation process in PPKTP. In our setup, a beta-barium borate (BBO) crystal is used to provide the required birefringence, whose cut angle is carefully selected to make sure that it cannot generate any nonlinear process. (**c**) In the timing link stabilization, the output of the link-locking PNS-BOC is sent to an MDL, a fiber stretcher (FS) and a PM erbium-doped optical fiber amplifier (EDFA) to compensate the long-term temperature drift, fast jitter imposed on the link and link power fluctuations, respectively. (**d**) Inside the FSC-BOMPD, a self-referenced signal (0.5M*f*_R_, *f*_R_ is the repetition rate of the optical input signal) is derived from the input optical pulse train to bias the Sagnac interferometer (SGI) at quadrature. The pulse train in the main SGI path performs electro-optical sampling of the RF input signal (N*f*_R_) to convert phase error into amplitude modulation. The modulated pulse train is detected and down-converted in-phase using another self-referenced signal (0.5* f*_R_) to baseband. The voltage phase error signal can be filtered by a proportional-integral controller and fed back to the microwave source for optical-to-microwave synchronization (alternatively, a feedback signal can also be applied to the laser of the optical input for microwave-to-optical synchronization). The zero-crossings of the microwave signal are then phase-locked to the pulse positions of the pulse train (i.e., Δ*θ*=0). (**e**) Out-of-loop characterization setups.

Taking the outcomes of this jitter analysis into account, an attosecond-precision laser-microwave network is demonstrated using the setup in Figure 3a. The timing signal from the master laser is distributed through a network that contains two independent fiber links of 1.2-km and 3.5-km length operated in parallel. The link outputs are used to synchronize a remote laser (e.g., serving as a pump-probe laser at the FEL end station in Figure 1) and a voltage-controlled oscillator (VCO; e.g., serving as a microwave reference of the FEL linear accelerator in Figure 1) simultaneously.

A beta-barium borate crystal with large birefringence is used in each locking BOC to realize a polarization-noise-suppressed BOC (PNS-BOC) for improved noise performance, as shown in Figure 3b. At the PNS-BOC output, since there are no time-dependent error voltages introduced by undesired pulse components (*E*_Ls_ and *E*_Rp_), each PNS-BOC can be locked exactly at the zero-crossing position of its BOC characteristic. This is crucial to make the BOC itself perfectly independent with laser amplitude noise so as to achieve attosecond precision. A feedback precision of ~2 as for the laser locking PNS-BOC is achieved with a low-noise BPD.

Residual SOD and TOD of links are compensated with additional dispersion-compensating fiber to suppress the link-induced Gordon–Haus jitter and to minimize the output pulse durations for high signal-to-noise ratio (SNR) in the BOCs. The link power is adjusted to minimize the nonlinearity-induced jitter, as well as to maximize the SNR for BOC locking. To eliminate power fluctuations caused by beam misalignment in the MDL, a feedback signal is sent to the erbium-doped optical fiber amplifier to control its pump current (Figure 3c).

In Figure 3d, a free-space-coupled BOMPD (FSC-BOMPD) is developed and employed for optical-to-microwave locking. The free-space components at the optical input can efficiently reduce long-term drifts caused by the environment, and the delay stages can enable precise phase tuning without backlash, microwave reflection and loss. Compared with other optical-microwave phase detectors^31, 32^, this new device is unaffected by optical input power fluctuations and can provide high SNR and a >10 × improvement in terms of long-term timing stability simultaneously (Supplementary Equations (S30–S37)), which are essential to achieve attosecond precision in the laser-microwave network.

Three characterization setups are adopted (Figure 3e): two timing link monitoring signals (TLM 1 and 2) are sent to an out-of-loop BOC to evaluate the link network performance; the master laser monitoring signal and the remote laser output signal (RLO) are sent to another BOC to characterize the remote laser synchronization; finally, the remote microwave output and RLO are compared with an out-of-loop FSC-BOMPD. The third setup is of great significance, since it directly measures the true relative timing jitter between a remotely synchronized mode-locked laser and a microwave source, which has never been shown before.

**Figure 4:**
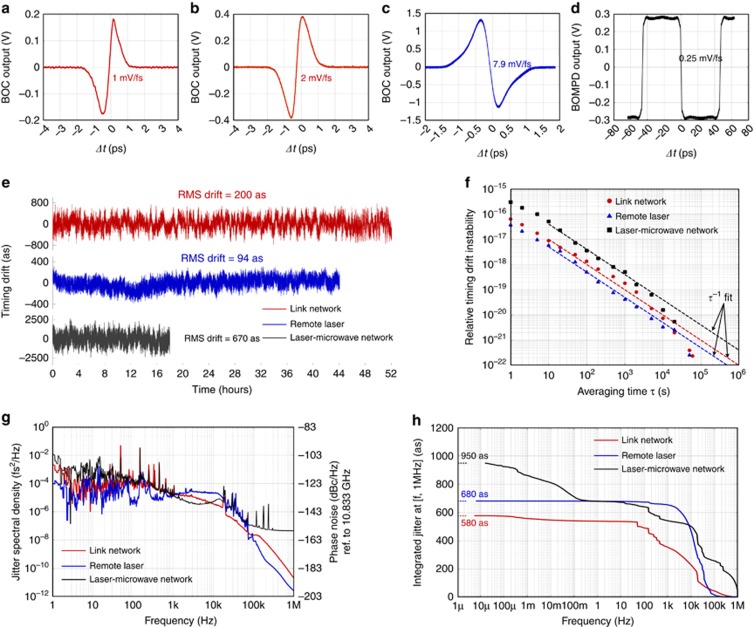
Measurement results of three characterization setups. The characteristics of link-locking PNS-BOC 1 and 2, laser locking PNS-BOC and VCO locking FSC-BOMPD are shown in (**a**–**d**), respectively. (**e**) Long-term timing drift (sampling rate=2 Hz). (**f**) Timing drift instability (Allan deviation) versus averaging time *τ*. A fit of *τ*^−1^ slope is shown. (**g**) Timing jitter spectral density and the corresponding phase noise referenced to 10.833 GHz at [1 Hz, 1 MHz]. (**h**) Integrated timing jitter.

The timing sensitivity of the link-locking PNS-BOC 1 and 2, laser locking PNS-BOC and VCO locking FSC-BOMPD are 1, 2, 7.9 and 0.25 mV fs^−1^, respectively (Figure 4a and 4d), which are large enough to support tight locking for the laser-microwave network. Stabilization of the 4.7-km link network is operated continuously for 52 h. The residual timing drift between TLM 1 and 2 <1 Hz is only 200-as RMS (Figure 4e, red curve); the relative timing drift instability is 6 × 10^−17^ with 1 s averaging time *τ* and reduced to 7.3 × 10^−21^ at *τ*=10^4^ s (Figure 4f, red circle). The equivalent phase noise at 10.833 GHz is <−110 dBc Hz^−1^ at 1 Hz and goes <−145 dBc Hz^−1^ after 20 kHz (Figure 4g, red curve); whereas the total integrated timing jitter from 6 μHz–1 MHz is only 580-as RMS (Figure 4h, red curve). Remote laser synchronization is achieved successfully for over 44 h without interruption. Residual timing drift is <100-as RMS (Figure 4e, blue curve), which is an order-of-magnitude improvement over previous results^27^, and corresponds to a relative timing instability of 2.5 × 10^−22^ in 50 000 s (Figure 4f, blue triangle). The integrated jitter is only 200 as in the range of 7 μHz–1 kHz and 680 as for 7 μHz–1 MHz (Figure 4h, blue curve). Finally, the whole laser-microwave network shows an unprecedented long-term precision of 670-as RMS out-of-loop drift over 18 h (Figure 4e, black curve). Compared with previous frequency-comb-based microwave transfer results^28^, this setup includes an additional fiber link and a remote laser synchronization system, yet it still achieves more than an order-of-magnitude improvement. The relative timing stability between the two remote-synchronized devices within the full frequency range from 15 μHz to 1 MHz is only 950-as RMS (Figure 4h, black curve). To the best of our knowledge, this is the first attosecond precision demonstration of remote optical-to-microwave synchronization, as well as the first demonstration of a synchronous laser-microwave network.

On the basis of the feedback model in the Supplementary Information, the out-of-loop jitter is contributed by the environmental noise imposed on the link, the electronic noise of the system, the master laser’s inherent jitter and the link-induced jitter (Supplementary Equation S2). In our experiment, most of the environmental noise is <1 kHz and can be well suppressed by the feedback loop. The link-induced jitter is also minimized by choosing the minimum link operating power required for tight link/laser/microwave locking. Therefore, the bumps from 1 to 20 kHz of all three curves in Figure 4g are mainly attributed to the master laser’s inherent jitter and the system electronic noise, which may even be amplified at those resonant frequencies of the feedback loop if not paid attention (Supplementary Fig. S2b and S2c). For the laser-microwave network results in Figure 4h (black curve), the power line noise at 50 Hz and its harmonics contribute ~250 as jitter, which can be removed by using cleaner power supplies. The residual drift <100 mHz is limited by the length fluctuations of the conventional coaxial cables in all RF paths of the FSC-BOMPDs, which can be improved by reducing all electronics into an integrated board or using special phase-stable cables with a much lower thermal-expansion ratio.

## Conclusion

In summary, by adopting new metrological timing detectors PNS-BOCs and FSC-BOMPDs, and reducing link-induced timing jitter and drift from nonlinear pulse propagation effects, long-term-stable attosecond timing precision has been achieved across a 4.7-km fiber-link network between remote optical and microwave devices. The attosecond precision laser-microwave network will enable next-generation FELs and other photon-science facilities to operate with the foreseen timing precision to unfold their full potential. This will drive new scientific efforts toward the making of atomic and molecular movies at the attosecond timescale, thereby opening up many new research areas in biology, drug development, chemistry, fundamental physics and material science. Besides, this technique will also accelerate developments in many other fields such as ultrastable clocks^33, 34^, gravitational wave detection^35^ and coherent optical antenna arrays^36^.

## Author contributions

FXK and ODM initiated the project. MX did the jitter limitation analysis and simulations. MX, KS and MYP contributed with the fiber network stabilization, and optical–optical synchronization system. MYP, AK and MX designed the FSC-BOMPD. MX, KS, AK and WW realized the laser-microwave network. All authors prepared the manuscript.
