# Supplementary material for: Attosecond precision multi-kilometer laser-microwave network
Source: Light Sci Appl. 2017 Jan 13;6(1):e16187–. doi: 10.1038/lsa.2016.187 (PMC6061888; doi:10.1038/lsa.2016.187)
Supplement: Supplementary Information [file lsa2016187x1.pdf]

Supplementary Information

Attosecond precision multi-km laser-microwave network

Ming Xin,[1*] Kemal Şafak,[1,2] Michael Y. Peng,[3] Aram Kalaydzhyan,[1] Wenting Wang,[1] Oliver D. Mücke,[1,2] and Franz X. Kärtner[1,2,3,*]

[1]*Center for Free-Electron Laser Science, Deutsches Elektronen-Synchrotron, Notkestrasse 85, Hamburg 22607, Germany*

[2]*Department of Physics, University of Hamburg and the Hamburg Center for Ultrafast Imaging, Luruper Chaussee 149, 22761 Hamburg, Germany*

[3]*Research Laboratory of Electronics, Massachusetts Institute of Technology, Cambridge, Massachusetts 02139, USA*

[*]Email: ming.xin@cfel.de, franz.kaertner@cfel.de

Here, we would like to provide the reader with more detailed information on the research presented in the main paper. First, the simulations investigating noise sources induced by the fiber link will be discussed. Then, the details of the fiber link network, remote laser synchronization and laser-microwave network experiments will be explained together with that of the FSC-BOMPD.

I. Simulation of link-induced noise

To investigate the link-induced noise, we have developed a feedback and numerical model of the timing link to simulate pulse timing jitter during nonlinear pulse propagation in the fiber link. The results show that even in the absence of environmental noise, residual link dispersion and nonlinearities add considerable excess jitter.

Feedback model of the timing link

Fig. S1 shows a flow diagram for the timing link feedback model. In the in-loop section, the detected timing jitter Y_{IL} by the in-loop BOC is first converted to voltage by the transfer function H_B, amplified by H_G with electronic noise E_B, and fed to the PI controller H_{PI} in a negative feedback configuration. The PI output, along with additive electronic noise E_{PI}, are amplified and converted back to timing delays J_C by H_{PZT}. Due to the round-trip propagation, J_C is added to the link delay twice. Furthermore, the round-trip link transmission time delay $2T_L$ needs to be considered for both J_C and master laser inherent jitter J_I. During link propagation, additional jitter from link-induced Gordon-Haus jitter[S1] and link-enhanced timing jitter due to fiber nonlinearities arises through H_{LR}. In practice, the environmental noise is usually significant below 1 kHz; therefore, the forward and backward link transmissions for a 3.5 km link impose the same environmental jitter value J_E on the link pulses. The in-loop jitter Y_{IL} is the relative jitter

between the round-trip link pulses and new pulses from the master laser. The out-of-loop jitter Y_{OL} is the relative jitter between the link output pulses and new pulses from the master laser. For the out-of-loop measurement, J_C and J_I experience a single-pass link propagation delay T_L. Furthermore, J_I experiences additional link-induced jitter via H_{LF}, and environmental jitter is contributed by J_E.

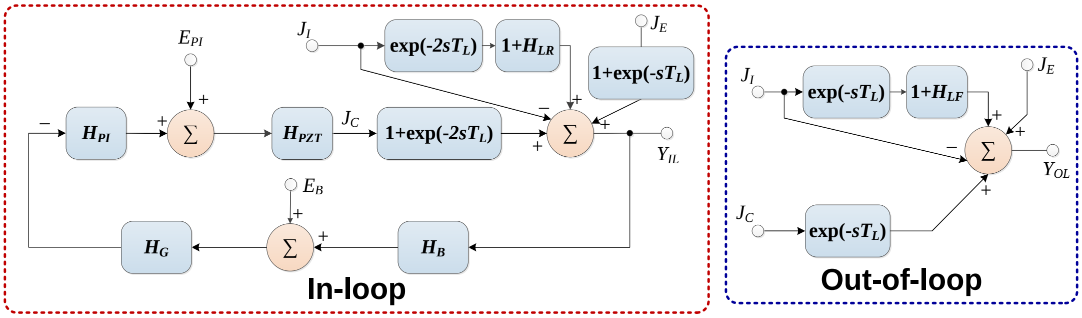

Figure S1. Feedback model of the timing link (J_I, inherent jitter of the master laser; J_E, integrated environmental jitter imposed on the link for single-trip link transmission; Y_{IL}, detected timing jitter by the in-loop BOC; H_B, transfer function of the in-loop BOC; E_B, electronic noise of the BPD in the in-loop BOC; H_G, transfer function of the amplifier in the BPD; H_{PI}, transfer function of the PI controller; E_{PI}, electronic noise of the PI controller; H_{PZT}, transfer function of the fiber stretcher and its driver; J_C, equivalent timing delay generated by the control loop for compensation; T_L, single-trip link transmission time; H_{LR}, equivalent transfer function of the link-induced time jitter for round-trip transmission; H_{LF}, equivalent transfer function of the link-induced timing jitter for forward link transmission; Y_{OL}, relative timing jitter between the link output pulses and the original pulses from the master laser).

Based on this model, we have

$$
\begin{aligned}
Y_{IL} &= J_I\left[\exp(-2sT_L)(1+H_{LR})-1\right]+J_C\left[1+\exp(-2sT_L)\right]+J_E\left[1+\exp(-sT_L)\right] \\
J_C &= \left[-(Y_{IL}H_B+E_B)G_BH_{PI}+E_{PI}\right]H_{PZT} \\
Y_{OL} &= J_I\left[\exp(-sT_L)(1+H_{LF})-1\right]+J_C\exp(-sT_L)+J_E
\end{aligned}
\qquad \text{(S1)}
$$

Then we obtain

$$
Y_{OL} = C_E J_E + C_N J_N + C_I J_I + C_L H_{LR} J_I \qquad \text{(S2)}
$$

where

$$
\begin{aligned}
J_N &= -\frac{E_B}{H_B} + \frac{E_{PI}}{H_B G_B H_{PI}} \\
C_E &= \frac{1+\mathbf{H}\left[1-\exp(-sT_L)\right]}{1+\mathbf{H}\left[1+\exp(-2sT_L)\right]} \\
C_N &= \frac{\mathbf{H}\exp(-sT_L)}{1+\mathbf{H}\left[1+\exp(-2sT_L)\right]} \\
C_I &= \frac{1+\mathbf{H}\left[1-\exp(-sT_L)\right]}{1+\mathbf{H}\left[1+\exp(-2sT_L)\right]}\left[\exp(-sT_L)-1\right] \\
C_L &= \frac{k+\mathbf{H}\left[k-\exp(-2sT_L)(1-k)\right]}{1+\mathbf{H}\left[1+\exp(-2sT_L)\right]}\exp(-sT_L) \\
\mathbf{H} &= H_B G_B H_{PI} H_{PZT} \\
k &= \frac{H_{LF} J_I}{H_{LR} J_I}
\end{aligned}
\qquad \text{(S3)}
$$

As Eq. (S2) indicates, the out-of-loop jitter Y_{OL} has four main contributions: the environmental noise imposed on the link, the electronic noise of the system, the master laser's inherent jitter and the link-induced jitter, with coefficients C_E, C_N, C_I and C_L, respectively, where k is a variable in the range [0, 1] that represents the degree of symmetry of link-induced jitter between the forward and backward link propagations. It should be noted that in our model, the environmental noise is treated as a discrete effect with a delay T_L between forward and backward trip. A more accurate approach is to divide the link into n-segments, and inject the environmental noise along different parts of the link, which will change C_E slightly.

The coefficients C_i, $i=E, N, I, L$ for a 3.5-km link can be calculated using the transfer functions of the experimental equipment. Fig. S2a-c show the calculated coefficients $|C_E|$, $|C_N|$, $|C_I|$, for five different PI controller gain settings. High gain is necessary to efficiently suppress the environmental noise below 1 kHz (Fig. S2a). However, the electronic noise from the BPD in the BOC and the PI controller rises with increasing gain (Fig. S2b). Furthermore, large gain peaks appear in Fig. S2a-c at frequencies $n/4T_L$ (n=1, 3, 5…) as well as around the resonant frequency of the fiber stretcher (about 18 kHz), if the feedback gain is too high. Therefore, to optimize the system performance, a medium gain needs to be adopted – see black curves in Fig. S2a-c. With this gain setting, $|C_I|$ in Fig. S2c exponentially increases from 0.02 at 1 kHz to 4.6 at about 16 kHz, which means that the master laser's inherent jitter can appear in the out-of-loop measurement through the feedback loop.

Also using such moderate gain value, $|C_L|$ with different k values is calculated in Fig. S2d. If the link-induced jitter from the forward and backward link transmission is almost

identical, like in the case of the Gordon-Haus jitter in Fig. 2a, then k is about 0.5, and $|C_L|$ is increased from 0.12 at 1 kHz to 2 at 14.6 kHz (Fig. S2d). In the case of the nonlinearity-induced jitter shown in Fig. 2b, if the backward link transmission power is much higher than for the forward path, the majority of the jitter is coming from the backward trip, k is almost 0. In Fig. S2d, $|C_L|$ is about 0.5 at 1 kHz and approach 1.5 at 16 kHz. On the other hand, if the forward power is greater than for the backward path, k is close to 1, and $|C_L|$ increases from 0.5 to 2.4 at [1 kHz, 14 kHz]. Above all, the link-induced jitter in Fig. 2a and b can transfer to the link-output through the feedback loop, either partially, completely or even with amplification.

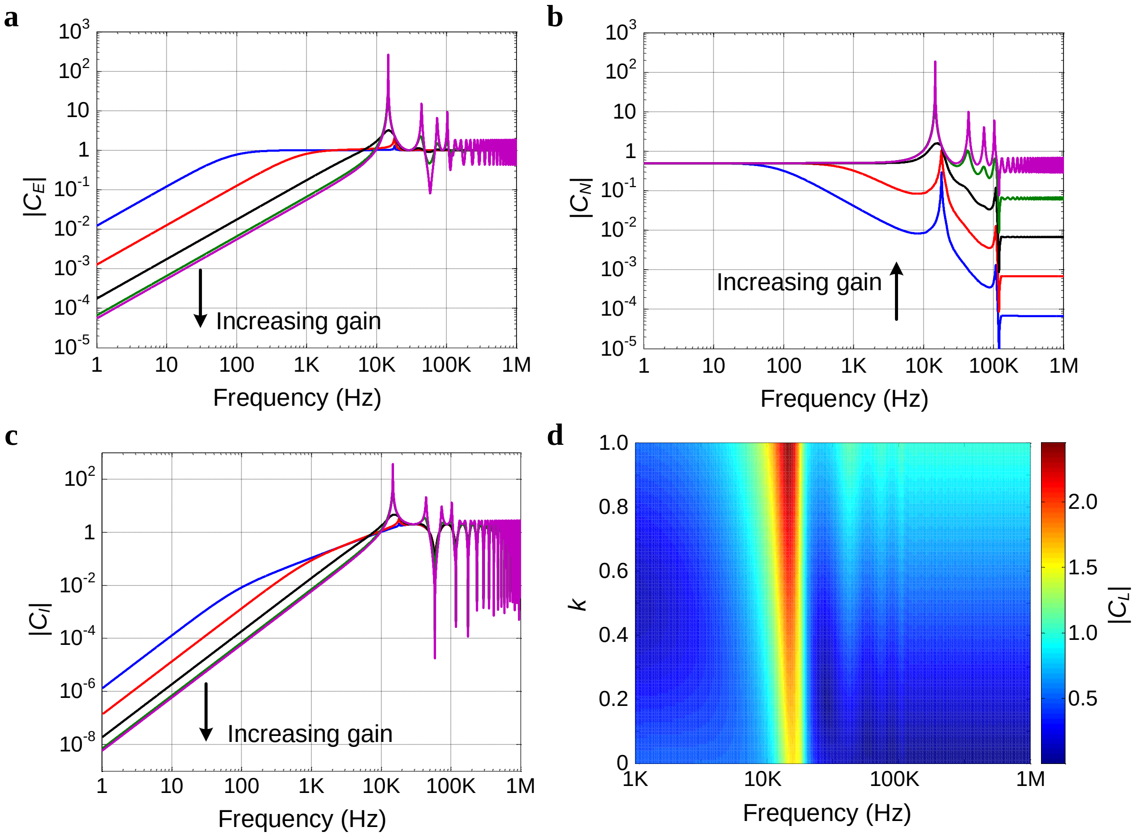

Figure S2. Simulation results for a 3.5-km timing link feedback model. **(a)** the coefficient for the environmental noise imposed on the link; **(b)** the coefficient for the electronic noise; **(c)** the coefficient for the master laser's inherent jitter; **(d)** the coefficient for the link-induced jitter with different k values.

Timing drift induced by link power fluctuations

An analytical model is developed to support the simulation results in Fig. 2c and 2d. The sum-frequency generation process in a BOC in undepleted-fundamental-frequency approximation is governed by the coupled wave equations[S2]:

$$\left[\frac{\partial}{\partial z}+\left(\frac{1}{v_1}-\frac{1}{v_3}\right)\frac{\partial}{\partial t}\right]E_1=0$$
$$\left[\frac{\partial}{\partial z}+\left(\frac{1}{v_2}-\frac{1}{v_3}\right)\frac{\partial}{\partial t}\right]E_2=0 \tag{S4}$$
$$\frac{\partial}{\partial z}E_3=i\frac{d_{eff}\omega_3}{n_3c}E_1E_2$$

where E_1, E_2 and E_3 are the electric fields of the link-reflected pulse, reference pulse and sum-frequency generation pulse, respectively, in the retarded time frame of E_3 with v_i denoting the group velocities of E_i (i=1, 2, 3). d_{eff} is the nonlinear optical coefficient, ω_3 is the carrier angular frequency of E_3, n_3 is the refractive index of E_3 in the crystal, and c is the vacuum speed of light. Pulse shaping due to dispersion in the crystal is not included. Based on Eq. (S4), we have

$$E_3(t)=K\int_0^{L_C}E_1(t-k_1z)E_2(t-k_2z)dz \tag{S5}$$

where L_C is the crystal length and

$$K=i\frac{d_{eff}\omega_3}{n_3c}$$
$$k_1=\frac{1}{v_1}-\frac{1}{v_3} \tag{S6}$$
$$k_2=\frac{1}{v_2}-\frac{1}{v_3}$$

If there is an initial delay T_D between E_1 and E_2, and L_C is sufficiently long, then Eq. (S5) can be approximated by

$$E_3(t,T_D)=K\int_0^{\infty}E_1(t-k_1z)E_2(t-k_2z-T_D)dz \tag{S7}$$

Suppose the reference pulse is so short that E_2 can be approximated by a Dirac delta function, then Eq. (S7) can be simplified as

$$E_3(t,T_D)=\begin{cases}KE_1(t-k_1\dfrac{t-T_D}{k_2}), & t>T_D\\ 0, & \text{else}\end{cases} \tag{S8}$$

The forward direction sum-frequency generation power of the BOC is

$$P_F(T_D) = \int_{-\infty}^{+\infty} \left|E_3(t, T_D)\right|^2 dt \tag{S9}$$

Based on Eqs. (S8) and (S9), we have

$$P_F(T_D) = \left|K\right|^2 \frac{k_2 - k_1}{k_2} \int_{T_D}^{\infty} \left|E_1(t)\right|^2 dt \tag{S10}$$

In first-order linear approximation, Eq. (S10) can be simplified to

$$P_F(T_D) = \begin{cases} \left|K\right|^2 \frac{k_2 - k_1}{k_2} E, & T_D < T_{D0} - \frac{1}{2}T_{BOC} \\ \left|K\right|^2 \frac{k_1 - k_2}{k_2} \frac{E}{T_{BOC}} \left(T_D - T_{D0} - \frac{1}{2}T_{BOC} \right), & T_{D0} - \frac{1}{2}T_{BOC} \le T_D \le T_{D0} + \frac{1}{2}T_{BOC} \\ 0, & T_D > T_{D0} + \frac{1}{2}T_{BOC} \end{cases} \tag{S11}$$

where T_{D0} is the zero-crossing time of the BOC, T_{BOC} is the linearly varying range of P_F, and

$$E = \int_{-\infty}^{+\infty} \left|E_1(t)\right|^2 dt \tag{S12}$$

The temporal center-of-gravity of E_1 is defined as the first order moment of t,

$$T_{COG} = \frac{1}{E} \int_{-\infty}^{+\infty} t\left|E_1(t)\right|^2 dt = \frac{1}{E} \lim_{T \to \infty} \int_{-T}^{T} t\left|E_1(t)\right|^2 dt \tag{S13}$$

Since

$$\begin{aligned} &\int_{-T}^{T} t\left|E_1(t)\right|^2 dt = t\int_{-\infty}^{t} \left|E_1(\tau)\right|^2 d\tau \Big|_{-T}^{T} - \int_{-\infty}^{+\infty} \int_{-\infty}^{t} \left|E_1(\tau)\right|^2 d\tau dt \\ &= -TE + \frac{k_2}{\left|K\right|^2 (k_2 - k_1)} \int_{-T}^{T} P_F(t) dt \end{aligned} \tag{S14}$$

Based on (S11), (S13) and (S14), we have

$$T_{COG} = T_{D0} \tag{S15}$$

Usually the pulse shape of E_1 is distorted during the link transmission. Now let us transfer to the retarded time frame of pulse propagation in the fiber link. Define $A(z, T)$ as

the slowly varying amplitude of the link pulse envelope. $A(z, T)$ is governed by the nonlinear Schrödinger equation[S3]:

$$\frac{\partial A}{\partial z}=\left(-\frac{\alpha}{2}-\frac{i\beta_2}{2}\frac{\partial^2}{\partial T^2}+\frac{\beta_3}{6}\frac{\partial^3}{\partial T^3}\right)A(z,T)+i\gamma\left(|A|^2 A+\frac{i}{\omega_0}\frac{\partial}{\partial T}(|A|^2 A)-T_R A\frac{\partial |A|^2}{\partial T}\right) \quad \text{(S16)}$$

where α is the fiber loss, β_2 is the second-order dispersion (SOD) coefficient, β_3 is the third-order dispersion (TOD) coefficient, γ is the nonlinear parameter for self-phase modulation (SPM), ω_0 is the carrier angular frequency, and T_R is the Raman parameter. The timing link consists of two fiber sections: standard polarization maintaining (PM) fiber, and PM dispersion compensating fiber (DCF). Here it is assumed that α and T_R are constant and β_2, β_3 and γ are z-dependent.

The slowly varying amplitude of the pulse envelope can be written in terms of a normalized amplitude function U on a time scale t normalized to the input pulse width T_0 as

$$A(z,T)=\sqrt{P_0}\exp(-\alpha z/2)U(z,t) \quad \text{(S17)}$$

$$t=\frac{T}{T_0} \quad \text{(S18)}$$

where P_0 is the peak power of the input pulse. Combining Eqs. (S16-18), $U(z,t)$ satisfies

$$U_z=-\frac{i\beta_2}{2T_0^2}U_{tt}+\frac{\beta_3}{6T_0^3}U_{ttt}+i\gamma P_0\exp(-\alpha z)\left(|U|^2 U+\frac{i}{\omega_0 T_0}(|U|^2 U)_t-\frac{T_R}{T_0}U|U|_t^2\right) \quad \text{(S19)}$$

where the subscripts t and z indicate partial derivatives, e.g., $U_z\equiv\partial U/\partial z$, $U_{tt}\equiv\partial^2 U/\partial t^2$.

The center-of-gravity of $A(z, T)$ can be written as

$$t_{COG}=\frac{\int_{-\infty}^{+\infty} T|A(T)|^2\,dT}{\int_{-\infty}^{+\infty}|A(T)|^2\,dT}=T_0\int_{-\infty}^{+\infty} t|U(t)|^2\,dt \quad \text{(S20)}$$

Based on Eq. (S15), in first-order linear approximation, the timing drift detected by the BOC can be characterized by changes in t_{COG}.

Using Eq. (S20) and (S19), we obtain

$$\frac{dt_{COG}}{dz} = T_0 \int_{-\infty}^{+\infty} t \left[\frac{i\beta_2}{2T_0^2}\left(U_{tt}^{*}U - U_{tt}U^{*}\right) + \frac{\beta_3}{6T_0^3}\left(U_{ttt}^{*}U + U_{ttt}U^{*}\right) - \frac{3\gamma P_0 \exp(-\alpha z)}{\omega_0 T_0} \left|U\right|_t^2 \left|U\right|^2 \right] dt \quad \text{(S21)}$$

At time positions far from a single pulse (t→±∞), it is reasonable to assume that the pulse temporal profile decays to zero and is well-behaved:

$$\begin{aligned} &\lim_{t\to\pm\infty} U(z,t) = 0 \\ &\lim_{t\to\pm\infty} \frac{\partial^n U}{\partial t^n} = 0, \quad n = 1, 2, \ldots \end{aligned} \quad \text{(S22)}$$

Utilizing Eq. (S22), Eq. (S21) can be rewritten as

$$\frac{dt_{COG}}{dz} = \frac{i\beta_2}{2T_0}\Omega + \frac{\beta_3}{2T_0^2}\Gamma + \frac{3\gamma P_0 \exp(-\alpha z)}{2\omega_0} S \quad \text{(S23)}$$

where

$$\begin{aligned} &\Omega = \int_{-\infty}^{+\infty} (U_t U^{*} - U U_t^{*}) dt \\ &\Gamma = \int_{-\infty}^{+\infty} \left|U_t\right|^2 dt \\ &\mathrm{S} = \int_{-\infty}^{+\infty} \left|U\right|^4 dt \end{aligned} \quad \text{(S24)}$$

The derivative of Ω and Γ with respect to *z* can be further calculated as follows

$$\begin{aligned} &\frac{d\Omega}{dz} = i2\gamma P_0 \exp(-\alpha z) \frac{T_R}{T_0} F_1(z) \\ &\frac{d\Gamma}{dz} = i\gamma P_0 \exp(-\alpha z) \left(F_2(z) - \frac{T_R}{T_0} F_3(z) \right) \end{aligned} \quad \text{(S25)}$$

where

$$\begin{aligned} &F_1(z) = \int_{-\infty}^{+\infty} \left(\left|U\right|_t^2 \right)^2 dt \\ &F_2(z) = \int_{-\infty}^{+\infty} \left|U\right|_t^2 (U U_t^{*} - U^{*} U_t) dt \\ &F_3(z) = \int_{-\infty}^{+\infty} \left|U\right|_{tt}^2 (U U_t^{*} - U^{*} U_t) dt \end{aligned} \quad \text{(S26)}$$

With numerical calculation, $F_2(z)$ and $F_3(z)$ can be easily verified to be nonzero for Gaussian and hyperbolic secant pulses with nonzero residual dispersion.

Substituting the integrated form of (S25) into (S23) and integrating (S23) over the link length L yields

$$\begin{aligned} t_{COG} &= \frac{1}{2T_0^2}\int_{-\infty}^{+\infty}\left|U_t(t,0)\right|^2 dt\int_0^L \beta_3 dz \\ &-P_0\left[\begin{array}{l} 2\frac{T_R}{T_0^2}\int_0^L \beta_2\int_0^z \gamma\exp(-\alpha z_1)F_1(z_1)dz_1dz+\frac{1}{T_0^2}\int_0^L \beta_3\int_0^z \gamma\exp(-\alpha z_1)F_2(z_1)dz_1dz \\ -\frac{T_R}{T_0^3}\int_0^L \beta_3\int_0^z \gamma\exp(-\alpha z_1)F_2(z_1)dz_1dz+\frac{3}{2\omega_0}\int_0^L \gamma\exp(-\alpha z)S(z)dz \end{array}\right] \end{aligned} \tag{S27}$$

Suppose that the input power with fluctuation $P_0(1+\delta)$ corresponds to a center-of-gravity with fluctuation $t_{COG}+\Delta t_{COG}$ for a given power fluctuation ratio δ. According to Eq. (S27), this yields

$$\frac{\Delta t_{COG}}{\delta} = -P_0\left[\begin{array}{l} 2\frac{T_R}{T_0^2}\int_0^L \beta_2\int_0^z \gamma\exp(-\alpha z_1)F_1(z_1)dz_1dz+\frac{1}{T_0^2}\int_0^L \beta_3\int_0^z \gamma\exp(-\alpha z_1)F_2(z_1)dz_1dz \\ -\frac{T_R}{T_0^3}\int_0^L \beta_3\int_0^z \gamma\exp(-\alpha z_1)F_3(z_1)dz_1dz+\frac{3}{2\omega_0}\int_0^L \gamma\exp(-\alpha z)S(z)dz \end{array}\right] \tag{S28}$$

The first term in the brackets on the right side of Eq. (S28) is dependent on the Raman response and SOD, whereas the second term is related to SPM and TOD. Furthermore, the third term corresponds to the Raman response and TOD, and the last term indicates the self-steepening effect. Hence, it can be concluded that the drift induced by power fluctuations is proportional to the input power level and is a combined effect of residual SOD, TOD, SPM, self-steepening and Raman response.

II. Fiber link network stabilization

The master laser used for the laser-microwave network operates with 1554.7-nm central wavelength, 170-fs pulse duration and 216.6675-MHz repetition rate, which is locked to a microwave reference with a 10-Hz feedback bandwidth to suppress long-term drifts. In practice, the laser power can be divided into several simultaneously operating timing links for the remote synchronization at different locations. Here, the laser power is split into two timing links. The input signal to each link stabilization setup is further divided into reference and link path pulses. The reference path lengths are set as short as possible (4 cm) to minimize timing errors introduced by environmental noise.

To assure that the forward and backward link transmission accumulates the same amount of jitter, the link pulse must travel along the same polarization axis during round-

trip propagation. Therefore, a 45° Faraday rotator before the fiber link is necessary to introduce a 90° round-trip polarization rotation to direct the reflected link pulse towards the BOC in the link stabilization block.

The BPDs of the link locking PNS-BOCs have two 1-MHz monitor outputs and a balanced output port with a 3-dB bandwidth of 100 MHz, while the BPD output bandwidth of the out-of-loop BOC 1 is 1 MHz.

Both links are constructed with a section of standard PM 1550 fiber, followed by a section of PM dispersion compensating fiber. The ratios of dispersion coefficients between the two fiber types are

$$\frac{\beta_{2d}}{\beta_{2p}} = -5.663$$
$$\frac{\beta_{3d}}{\beta_{3p}} = -5.613 \qquad \text{(S29)}$$

where β_{2p} and β_{3p} are the SOD and TOD coefficient of PM 1550, and β_{2d} and β_{3d} are the SOD and TOD coefficient of PM dispersion compensating fiber. Due to this ratio difference, it is not possible to compensate SOD and TOD simultaneously. For example, if the SOD of the 3.5-km link is completely eliminated, 26-m worth of TOD from PM 1550 would be uncompensated. An optical auto-correlator is used to confirm the temporal duration of the link pulses. Due to the residual TOD, pulse durations of ~400 fs are obtained for both links. Based on Fig. 2c, this residual TOD can introduce a drift of at least 2 fs for ±5% link power fluctuations. Link power stabilization in Fig. 3c is therefore indispensable to achieve attosecond drift.

A detailed schematic of the link network characterization setup is given in Fig. S3. Two polarizers are placed before and after the link to improve the polarization extinction ratio. The output voltage of the PI controller is divided into two paths. The first path compensates for fast noise in the link and consists of a high-voltage amplifier that drives a PM fiber stretcher. The second path compensates for long-term environmental drift and consists of a data acquisition card (DAQ) card that samples the timing error and controls the MDL through a Labview program. Link power stabilization is achieved by measuring the BPD monitor ports in the PNS-BOCs and feeding back to the pump current of the EDFA through a TCP/IP network. While the timing drift due to power fluctuations in the forward link transmission can be neglected (because the link power is relatively low), the power stabilization scheme is necessary to remove the excess timing drift in the reverse link transmission after EDFA amplification for improved out-of-loop performance.

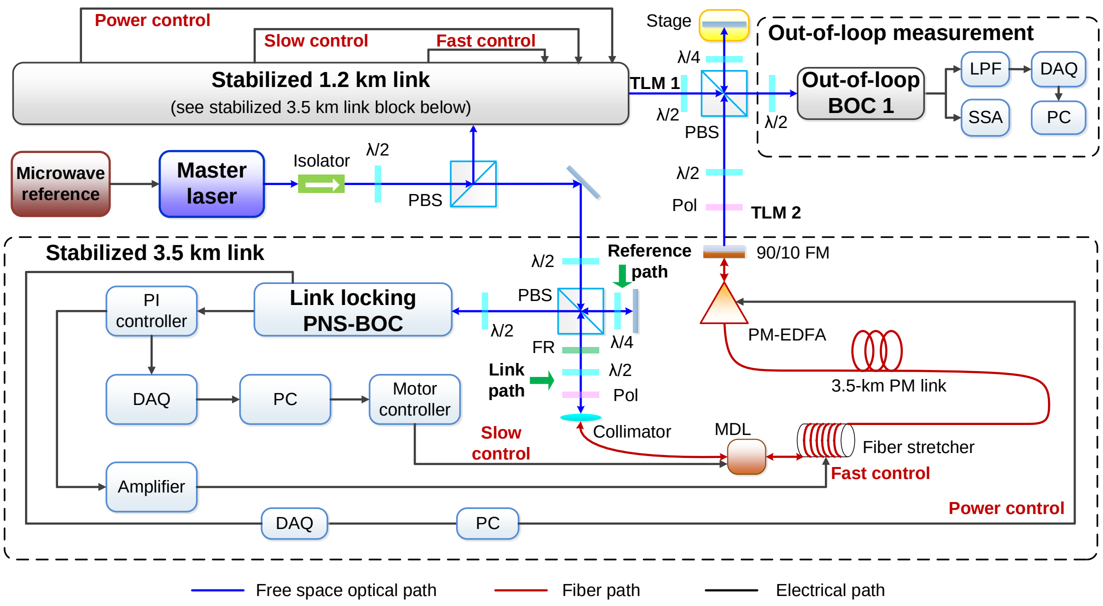

Figure S3. Detailed scheme of the link network stabilization (PBS, polarization beam splitter; λ/2, half-wave plate; λ/4, quarter-wave plate, FR, 45 °Faraday rotator; Pol, polarizer; 90/10 FM, 90/10 transmission/reflection fiber mirror; LPF, 1-Hz low pass filter; SSA, signal source analyzer; PC, computer).

To minimize timing errors resulting from thermally-induced length fluctuations in the reference paths, all free-space optics are mounted on a temperature-stabilized breadboard with a Super-Invar surface sheet. With temperature fluctuations controlled below ±0.05 K, the effective timing-instability of free-space beam paths due to thermal expansion is ±1 as/cm. Lead foam is placed beneath the setup to damp table vibrations. A two-layer enclosure with acoustic heavy foil for the inner layer and high-density polyethylene (HDPE) for the outer layer is built to provide acoustic isolation for all optical components, except for the two fiber links which are placed outside of the enclosure and subjected to environmental changes.

Fig. S4 shows the monitored results during the link network stabilization demonstration. During 52 hours, about 8.6 ps and 35 ps link drift are compensated by the MDLs in the 1.2 km and 3.5 km long links, respectively. Temperature and relative humidity on the fiber links changed by 0.3 K and 4% respectively. With the help of power feedback control, power fluctuations in the 1.2-km link are suppressed to within ±0.2%. Due to the coarse resolution of the EDFA pump current, the power fluctuations in the 3.5-km link can only be stabilized to within ±1% and serves as the main drift contribution in Fig. 4h (red curve) from 300 μHz to 1 Hz.

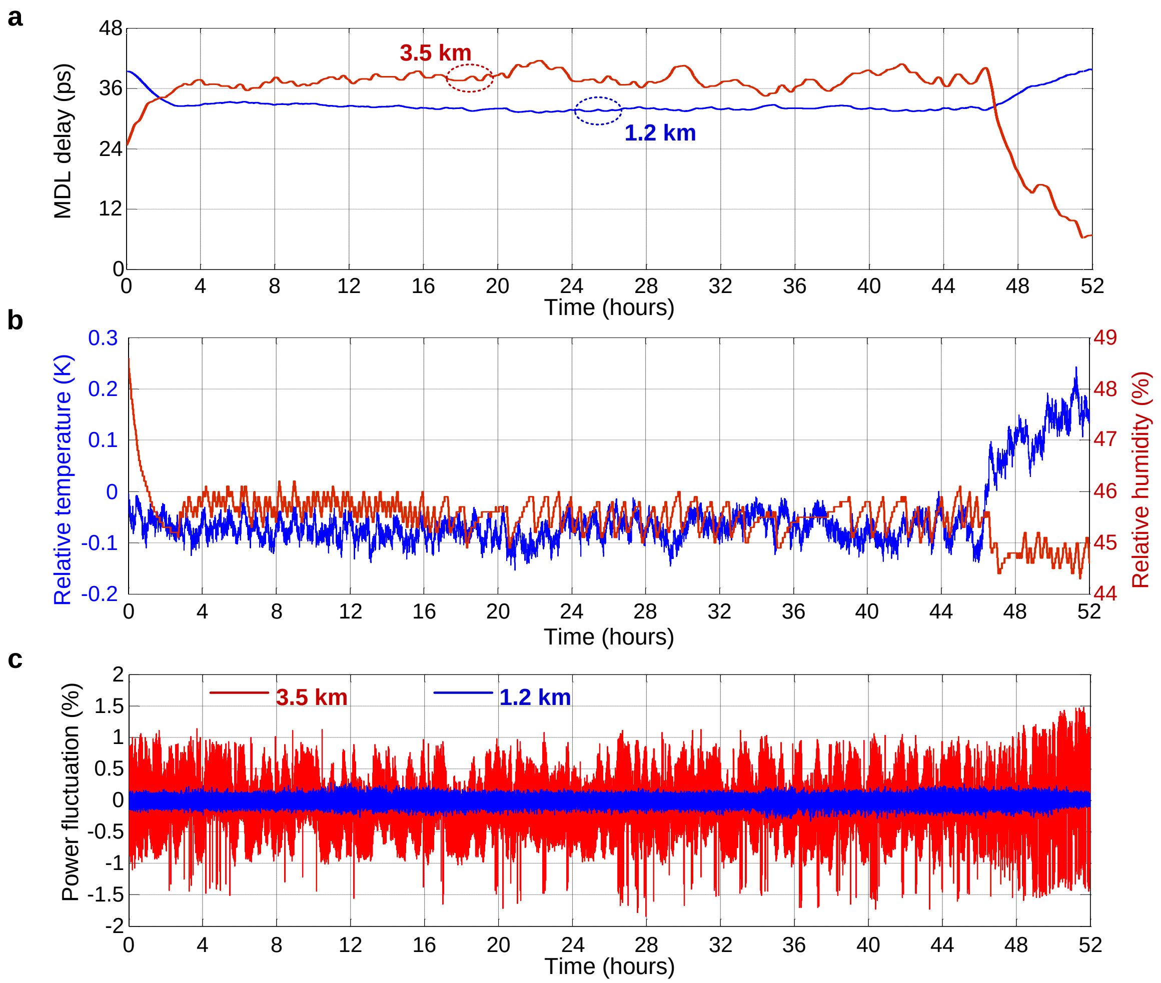

Figure S4. Monitored signals in a link network stabilization experiment. **(a)** Compensated link drift by the MDLs; **(b)** environmental temperature and humidity change; **(c)** power fluctuation after power control.

III. Remote laser synchronization

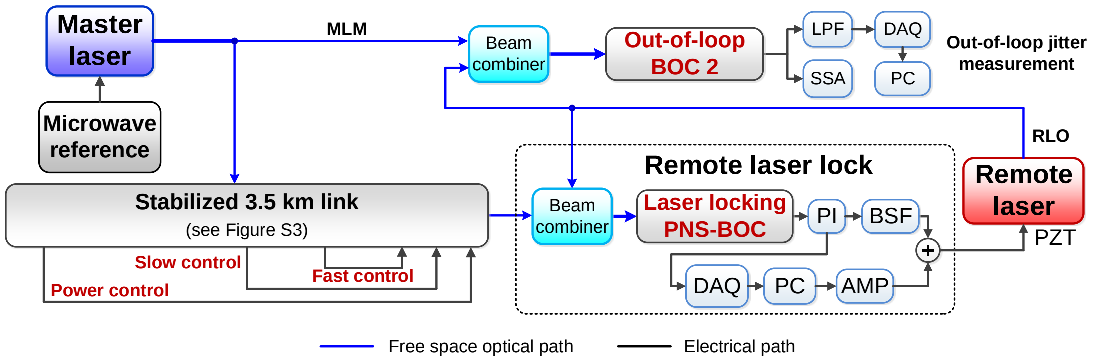

Figure S5. Detailed schematic of the remote laser synchronization (BSF, band stop filter; PZT, piezoelectric transducer).

In Fig. 3a, the remote laser operates at 1553.4-nm central wavelength with 170-fs pulse width and 216.6685-MHz repetition rate. Pulse trains from the 3.5 km link output and the remote laser are combined with orthogonal polarization and detected by the laser-locking PNS-BOC with a timing sensitivity of 7.9 mV/fs. As shown in Fig. S5, the laser-locking PNS-BOC output voltage is filtered by a PI controller. Due to the limited bandwidth of the amplifier, the PI output is separated into two paths to optimize feedback control for slow and fast noise independently. The first path is sampled by a data acquisition card and analyzed by a Labview program to generate a DC offset voltage to compensate slow timing drifts. A high-gain amplifier is used to extend the compensation range. The second path, which compensates fast noise, is not amplified to minimize phase excursions at high offset frequencies to achieve high locking bandwidth; a band stop filter is used to eliminate potential feedback loop resonances. The voltage summer output recombines the two paths to drive the piezoelectric transducer (PZT) of the remote laser, which has a sensitivity of 17.4 Hz/V.

During the 44-hour operation of remote laser synchronization (see Fig. S6), the link stabilization compensates about 61-ps link drift while the remote laser synchronization corrects for a 400-Hz repetition rate drift of the remote laser. The temperature variation is about 0.6 K and the relative humidity change is about 4% (measured on the fiber link), which are typical laboratory environmental conditions.

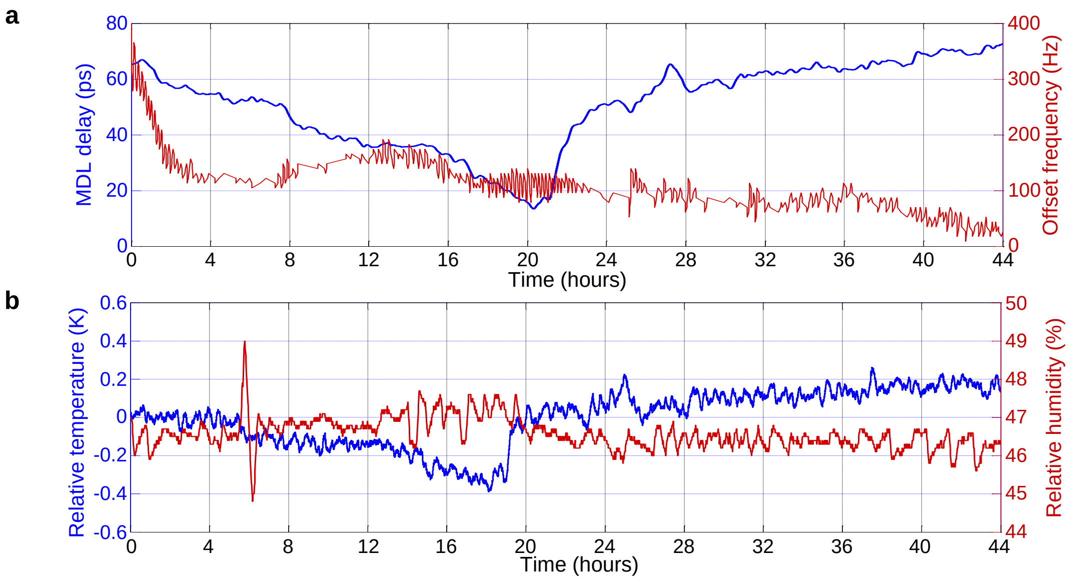

Figure S6. Monitoring results in a remote laser synchronization experiment. **(a)** Compensated drift by the MDL and the PZT offset frequency; **(b)** temperature and humidity change of the fiber link.

IV. Free-space-coupled balanced optical-microwave phase detector

To improve the timing stability, we developed a new FSC-BOMPD, as shown in Fig. S7. Key characteristics in this BOMPD architecture are summarized as follows: first, free space components, such as PBS, half-wave plates and quarter-wave plates are used before the SGI, and the total length of the SGI is spliced to be as short as possible. This efficiently reduces the long-term drifts caused by the environment. Second, high-frequency (multi-GHz) modulation in the phase modulator is enforced to ensure unidirectional phase modulation. This eliminates the repetition-rate dependence of the SGI, thus improving its robustness and long-term stability. Third, error signal demodulation is performed at the lowest possible frequency to maximize SNR at photo detection and to minimize thermally induced phase changes in the RF signal paths for long-term stability. Fourth, free space delay stages are used to control the relative delay between different paths; this enables precise phase tuning without backlash, microwave reflection and loss when compared with RF phase shifters. Lastly, AM- and PM-sensitive signal paths are optimized to achieve an AM-PM suppression ratio of -50 dB, which is a high figure-of-merit for optical-to-microwave conversion.

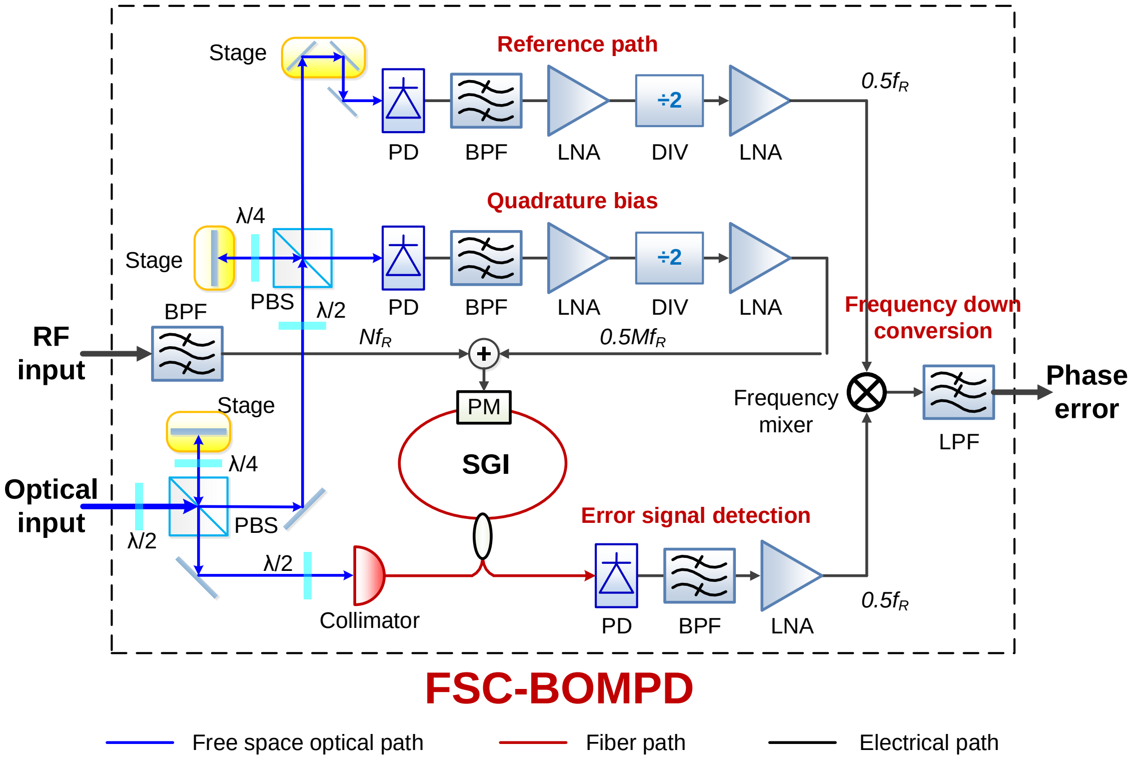

Figure S7. Detailed scheme of the free-space-coupled balanced optical-microwave phase detector (FSC-BOMPD) (BPF, bandpass filter; LPF, lowpass filter; PD, photodetector; LNA, low-noise amplifier; DIV, frequency divider; PM, phase modulator; f_R, the repetition rate of the optical input pulse train).

Compared with other BOMPD structures[S4], our FSC-BOMPD is insensitive to optical input power fluctuations. Besides that, phase fluctuations from the bias and reference

paths also cannot affect the phase error output. So it can accurately measure the phase difference between the RF and optical input. An analytic derivation of the output voltage of the FSC-BOMPD is given below. To avoid ambiguity, all the symbols in the following equations are independent with those in Eqs. (S1-29). If the optical input pulse is sufficiently short, the optical pulse train power at the SGI input can be approximated by

$$P_{in}(t) = P_a T_R \sum_{n=-\infty}^{\infty} \left(1+\Delta_{RIN}(t)\right) \delta(t - nT_R - \Delta_J(t)) \tag{S30}$$

where $\delta(t)$ is a Dirac function, $T_R = 1/f_R$ is the period of the pulse train, P_a, $\Delta_{RIN}(t)$ and $\Delta_J(t)$ are the average power, power fluctuation and timing jitter of the pulse train, respectively.

The driving signal of the phase modulator can be written as

$$\varphi(t) = \Phi_0 \sin\left[2\pi f_0 \left(t+\Delta t_0\right)\right] + \Phi_b \sin\left[2\pi (M+0.5) f_R \left(t+\Delta t_b\right) + \Delta\phi\right] \tag{S31}$$

where Φ_0, f_0 and Δt_0 are the amplitude, frequency and timing jitter of the RF input signal, respectively. Φ_b is the amplitude of the RF signal from the quadrature bias path. $\Delta\phi$ and Δt_b are the fixed relative phase and relative timing jitter between the pulse train and the RF bias signal, respectively. M is an integer.

After circulating in the SGI, the output optical power can be expressed as

$$\begin{aligned} P(t) &= (1-\alpha) P_{in}(t) \sin^2(\varphi/2) \\ &= (1-\alpha) P_a T_R \times \\ &\sum_{n=-\infty}^{\infty} \sin^2\left[\frac{1}{2}\Phi_0 \sin\left[2\pi f_0 t + \theta_e\right] + \frac{1}{2}\Phi_b \sin\left[2\pi (M+0.5) f_R t + \theta_b + \Delta\phi\right]\right] \left(1+\Delta_{RIN}\right) \delta(t - nT_R) \end{aligned} \tag{S32}$$

where α is the loss of the SGI, $\theta_e = 2\pi f_0(\Delta t_0 + \Delta_J)$ is the relative phase error between the SGI and RF input signals, and $\theta_b = 2\pi(M+0.5) f_R \Delta t_b$ is the phase fluctuation of the bias path.

Suppose the frequency of the RF and optical input signals are locked with each other by the FSC-BOMPD, then $f_0 = N f_R$, N is an integer. Since $\delta(t-nT_R)$ is nonzero only if $t=nT_R$, and θ_e, θ_b <<1, Eq. (S32) can be simplified as

$$P(t) \approx \frac{1}{2}(1-\alpha) P_a T_R \sum_{n=-\infty}^{\infty} \left[1 - \cos\left(\Phi_0 \theta_e + (-1)^n \Phi_b \sin(\Delta\phi + \theta_b)\right)\right] \left(1+\Delta_{RIN}\right) \delta(t - nT_R) \tag{S33}$$

Let

$$\Phi_b \sin(\Delta\phi) = \pi/2 \tag{S34}$$

Eq. (S33) can be written as

$$P(t)=\frac{1}{2}(1-\alpha)P_aT_R\sum_{n=-\infty}^{\infty}\left[1+\Phi_b\left(-\frac{1}{2}\sin\Delta\phi\theta_b^2+\cos\Delta\phi\sin\theta_b\right)\right]\left(1+\Delta_{RIN}\right)\delta(t-nT_R)$$
$$+\frac{1}{2}(1-\alpha)P_aT_R\sum_{n=-\infty}^{\infty}\Phi_0\theta_e\left(1+\Delta_{RIN}\right)\delta(t-nT_R)e^{j\pi f_R t} \quad \text{(S35)}$$

In the frequency domain, the first item on the right side of Eq. (S35) represents the components at Kf_R, while $(K+1/2)f_R$ for the second item (K is an integer). Since θ_b only appears in the first item, the phase fluctuation from the bias path cannot affect the down-conversion signal at $f_R/2$.

Assume the RF output signal from the reference path is

$$V_r=\Phi_R\sin\left(\pi f_R(t+\Delta t_r)\right) \quad \text{(S36)}$$

where Φ_R is the amplitude of the reference signal, and Δt_r is the relative timing jitter between the reference path and the SGI path. Then the error signal after down conversion is

$$V_e=\frac{C}{2}(1-\alpha)P_aT_RV_r\left(1+\Delta_{RIN}\right)\cos\left(\pi f_R\Delta t_r\right)\Phi_0\theta_e \quad \text{(S37)}$$

where C is a constant coefficient related to the mixer, low-pass filters and other RF components in the down conversion path. If f_R=216 MHz, 3 mm length change (10 ps) from the reference path can only introduce 2×10^{-5} change to V_e. So the phase fluctuation of the reference path contributes little to the output error signal. Similarly, since usually Δ_{RIN} <<1, the effect due to optical input power fluctuations Δ_{RIN} is also negligible. So V_e is mainly determined by θ_e, the relative timing jitter between the RF and SGI input signals. The free-space optical paths before the SGI are well isolated from environmental changes, so that the FSC-BOMPD can accurately detect the timing jitter between the RF and optical input signals without introducing systematic errors.

V. Synchronous laser-microwave network

A detailed schematic of the laser-microwave network characterization setup is given in Fig. S8, that refers to Figs. S3, S5 and S7. A sapphire-loaded crystal oscillator (SLCO) serves as the remote VCO source. It operates at a 10.833-GHz center frequency, which is an integer multiple (×50) of the master laser repetition rate. A low-phase-noise amplifier (LPNA) after the SLCO is used to maximize the phase discriminant of the FSC-BOMPDs. In each FSC-BOMPD (see Fig. S7), the optical input is first separated into the SGI, bias and reference paths with free space PBSs. The pulse train in the bias path is detected, filtered at the 59th harmonic of the repetition rate, frequency-divided to 6.37 GHz (=29.5f_R), and adjusted in amplitude and phase to achieve Eq. (S34) for quadrature bias. Using similar detection electronics, the reference signal is generated at 108 MHz (=0.5f_R).

When the input RF signal is applied to SGI, any phase error between the input signals will induce amplitude modulation in the pulse train at the SGI output. The error modulation sidebands of the pulse train are directly detected, filtered at 108 MHz and down-converted in-phase with the 108-MHz reference signal to generate the output phase error signal at baseband. The phase error voltage signal in the VCO locking FSC-BOMPD is fed back to the frequency tuning port of the SLCO to achieve remote optical-to-microwave synchronization through the 1.2-km link. The out-of-loop FSC-BOMPD compares the relative timing error between the remote laser and the SLCO, to evaluate the performance of the whole synchronization network.

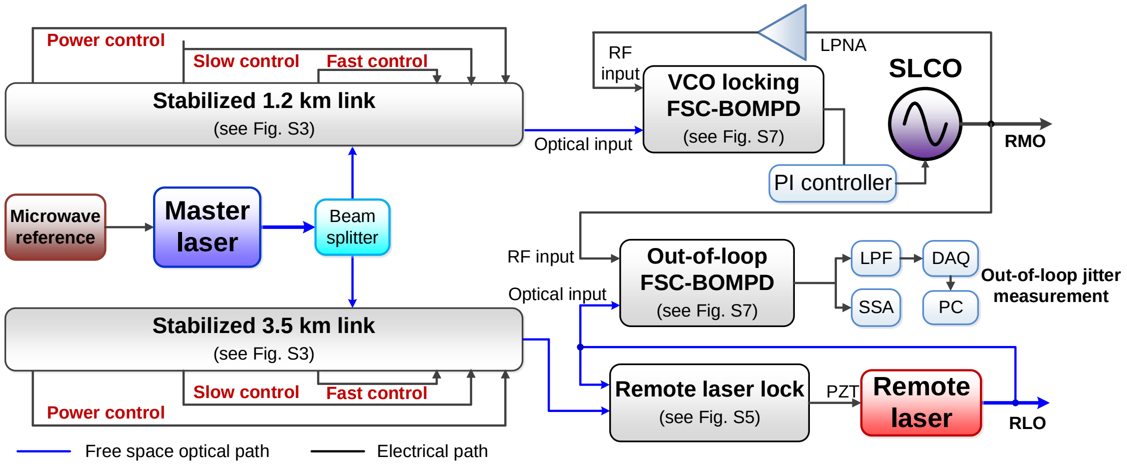

Figure S8. Detailed scheme of laser-microwave network synchronization.

To avoid fiber nonlinearities in the 1.2-km link, input optical power into the VCO locking FSC-BOMPD is about +9 dBm: both bias and reference arms utilize 0 dBm while the SGI obtains +5 dBm after collimator loss. For the out-of-loop FSC-BOMPD, due to all-free-space optics in the remote laser part, there is no nonlinearity onset and totally +14 dBm is provided to the optical input: 0 dBm for bias and reference paths and +10 dBm for SGI after collimator, respectively. The RF input power for each FSC-BOMPD is about +21 dBm. The phase sensitivity of the VCO locking and out-of-loop FSC-BOMPDs are about 0.25 mV/fs and 2.5 mV/fs, respectively. In Fig. 4g (black curve), The 20-kHz locking bandwidth is limited by the excitation of higher-order poles and zeroes due to high feedback gain. In some parts of the FSC-BOMPDs, 1 fs of the phase difference translates to a voltage shift of only several micro-volts. Therefore, all digital circuits are galvanically isolated from the FSC-BOMPDs, while the analog power lines are connected in a single point configuration through electromagnetic interference filters to eliminate undesirable ground loops and noise pick-ups and prevent electrostatic discharge. Thus, robust functioning of the FSC-BOMPD in actual service has been achieved without dropping out of lock during several weeks of operation. The FSC-BOMPDs have no active temperature control system; however, they are placed within a closed thermal insulated chamber to damp temperature and humidity changes of the

environment. After the necessary warm-up time, the two FSC-BOMPDs do not experience more than 0.08 K of temperature drift and 0.6% relative humidity change during the measurement period (Fig. S9).

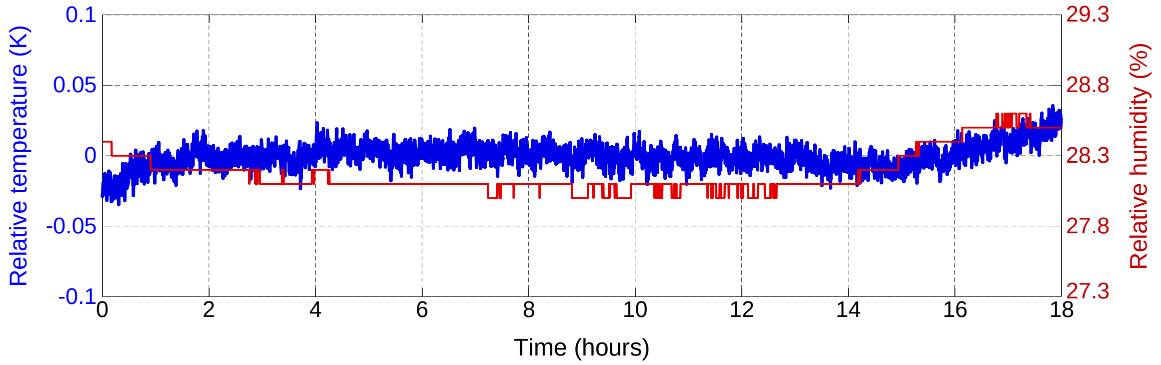

Figure S9. The environmental temperature and humidity change of the two FSC-BOMPDs in laser-microwave network characterization.

Reference

S1. Gordon JP, Haus HA. Balanced Random walk of coherently amplified solitons in optical fiber transmission. *Opt Lett* 1986; **11**: 665-667.

S2. Diels JC, Rudolph W. *Ultrashort Laser Pulse Phenomena.* Academic Press, 2006.

S3. Agrawal GP. *Nonlinear Fiber Optics.* Academic Press, 2012.

S4. Jung K, Kim J. Sub-femtosecond synchronization of microwave oscillators with mode-locked Er-fiber lasers. *Opt Lett* 2012; **37**: 2958-2960.
